# Supplementary material for: Glucocorticoid Receptor Gene (NR3C1) Polymorphisms and Metabolic Syndrome: Insights from the Mennonite Population
Source: Genes (Basel). 2023 Sep 15;14(9):1805. doi: 10.3390/genes14091805 (PMC10530687; doi:10.3390/genes14091805)
Supplement: Supplementary file 1 [file genes-14-01805-s001.zip › Table S2.pdf]

Table S2. Allele distributions of *NR3C1* polymorphisms investigated in this study.

NR3C1 genetic variant	Mennonite	European Non Finish	Mennonite vs. European Non Finish	Amish	Mennonite vs. Amish	Brazilian	Mennonite vs. Brazilian
	n/N total (frequency)	n/N total	*p*corr	n/N total	*p*corr	n/N total	*p*corr
rs258763 A>T	351/602 (0.583)	38374/67970	0.12	585/906	0.074	1065/2342	**7.4×10^{-11}**
rs6196 T>C	130/650 (0.2)	10879/68000	**0.011**	221/912	**0.049**	288/2342	**4.3×10^{-6}**
rs926407137 G>A	10/650 (0.015)	0/66766	**1.9×10^{-20}**	0/908	**2.3×10^{-4}**	NA	-
rs17209258 T>C	119/650 (0.18)	14468/67838	0.1	258/910	**1.5×10^{-5}**	410/2342	0.64
rs34176759 TA>T	69/650 (0.108)	14394/67950	**9.6×10^{-12}**	254/906	**1.1×10^{-16}**	401/2342	**6.6×10^{-5}**
rs33944801 G>C	67/650 (0.105)	14394/67928	**1.7×10^{-12}**	254/908	**2.9×10^{-17}**	401/2342	**2.9×10^{-5}**
rs258813 C>T	272/650 (0.418)	21892/67952	**5.8×10^{-7}**	302/912	**5.3×10^{-4}**	642/2342	**1.9×10^{-11}**
rs761295829 C>T	2/650 (0.003)	0/68018	**2.7×10^{-4}**	0/912	0.26	NA	-
rs6188 G>T	272/650 (0.274)	21933/67972	**5.9×10^{-7}**	301/910	**4.4×10^{-4}**	642/2342	**1.4×10^{-11}**
rs56149945 A>G	27/650 (0.041)	2244/68032	0.22	1/912	**1.9×10^{-9}**	34/2342	**9.6×10^{-5}**
rs6190 G>A	35/650 (0.054)	1863/68046	**1.3×10^{-4}**	1/912	**1.8×10^{-12}**	31/2342	**2.6×10^{-8}**
rs6189 G>A	35/650 (0.054)	1863/68046	**1.3×10^{-4}**	1/912	**1.8×10^{-12}**	31/2342	**2.6×10^{-8}**
rs192978343 T>G	4/650 (0.006)	399/68032	0.80	4/912	0.80	3/2342	0.13
rs6877893 C>T	261/600 (0.435)	31437/67914	0.086	352/908	0.16	1235/2342	**5.5×10^{-6}**
rs41423247 C>G	220/602 (0.365)	25257/67930	0.91	478/910	**3.3×10^{-9}**	685/2342	**6.4×10^{-5}**
rs10482614 G>A	130/650 (0.2)	10607/67986	**0.004**	220/910	0.056	279/2342	**9.1×10^{-7}**
rs571795102 A>G	23/650 (0.035)	178/67930	**1.2×10^{-17}**	0/912	**1.4×10^{-9}**	4/2342	**4.4×10^{-12}**
rs10482606 T>C	3/650 (0.004)	380/67832	1.0	4/910	1.0	3/2328	0.37
rs10482605 T>C	142/650 (0.218)	11398/67686	**0.0014**	81/912	**3.7×10^{-12}**	362/2342	**3.7×10^{-4}**
rs5871845 G>GC	44/650 (0.067)	3799/67956	0.3	34/912	**0.027**	NA	**-**
rs3806854 T>C	124/648 (0.19)	10337/64314	**0.041**	213/864	**0.019**	278/2342	**1.1×10^{-5}**
rs3806855 T>G	125/648 (0.19)	10356/65778	**0.025**	213/880	**0.025**	278/2342	**7.6×10^{-6}**

Table S2. *cont*

rs1192533423 T>G	26/650 (0.04)	503/37336	**6.0×10^{-6}**	19/494	1.0	NA	**-**
rs7701443 T>C	242/592 (0.408)	27510/67962	0.76	309/912	**0.027**	1076/2342	**0.016**
rs72802813 C>T	116/604 (0.192)	12965/67898	0.96	107/912	**3.2×10^{-4}**	444/2342	0.96

In bold: significant. P-value was corrected by Benjamini & Hochberg method. n: number of allele count; NA: no data available.
