# Supplementary material for: Glucocorticoid Receptor Gene (NR3C1) Polymorphisms and Metabolic Syndrome: Insights from the Mennonite Population
Source: Genes (Basel). 2023 Sep 15;14(9):1805. doi: 10.3390/genes14091805 (PMC10530687; doi:10.3390/genes14091805)
Supplement: Supplementary file 1 [file genes-14-01805-s001.zip › Tables S1.pdf]

Table S1. PCR and pyrosequencing primers.

Amplification primer (PCR)	
Forward	5′-GTTTTTAATTTTTTCCTAAAACCTG-3′
Reverse	5′-biotin-CCTTCCCTAAAACCTCCC-3′
Sequencing primer (pyrosequencing)	
Seq 1 – 35 to 39 CpG	5′-ATTTTTTCCTAAAACCTGT-3′
Seq 2 – 40 CpG	5′-ATTTGGTTTTTTTGGGG-3′
Seq 3 – 43 to 47 CpG	5′-TTTAGTTTTTTTTTT-3′
