# Supplementary figures and images for: Glucocorticoid Receptor Gene (NR3C1) Polymorphisms and Metabolic Syndrome: Insights from the Mennonite Population
Source: Genes (Basel). 2023 Sep 15;14(9):1805. doi: 10.3390/genes14091805 (PMC10530687; doi:10.3390/genes14091805)

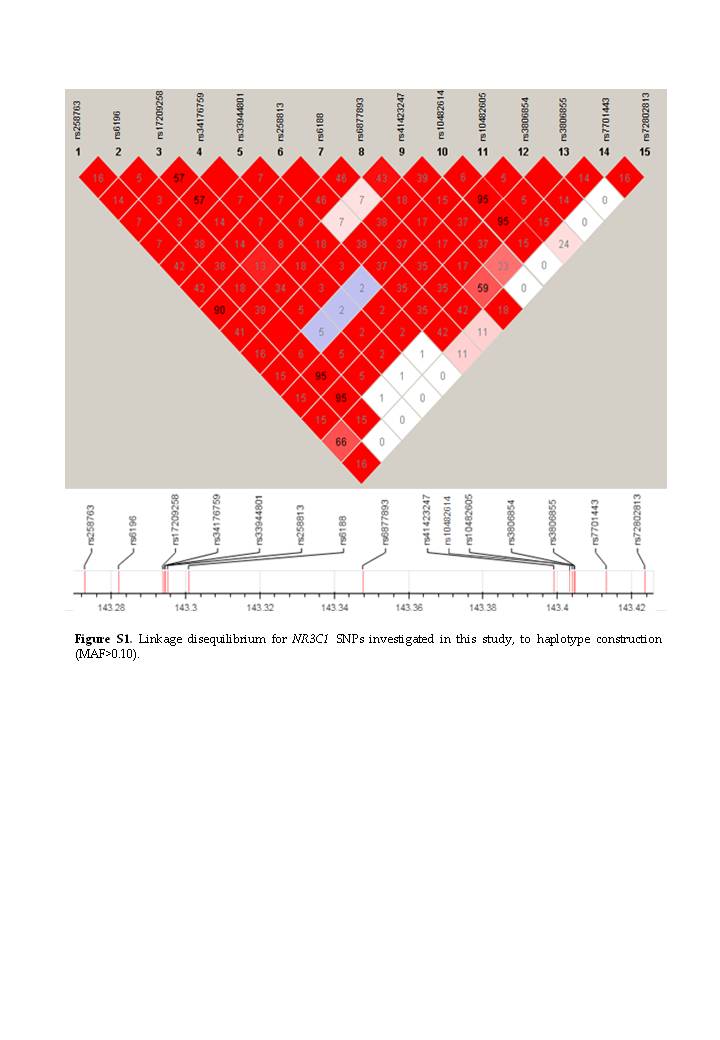

Supplement: Supplementary file 1 [file genes-14-01805-s001.zip › Figure S1_300dpi.jpg]

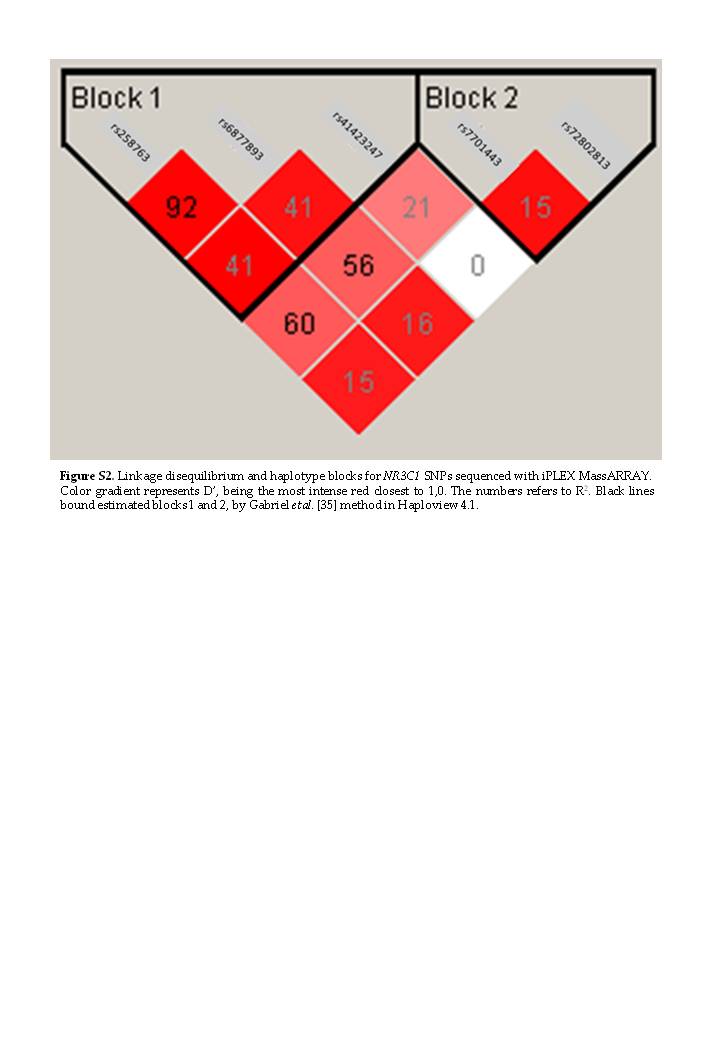

Supplement: Supplementary file 1 [file genes-14-01805-s001.zip › Figure S2_300dpi.jpg]

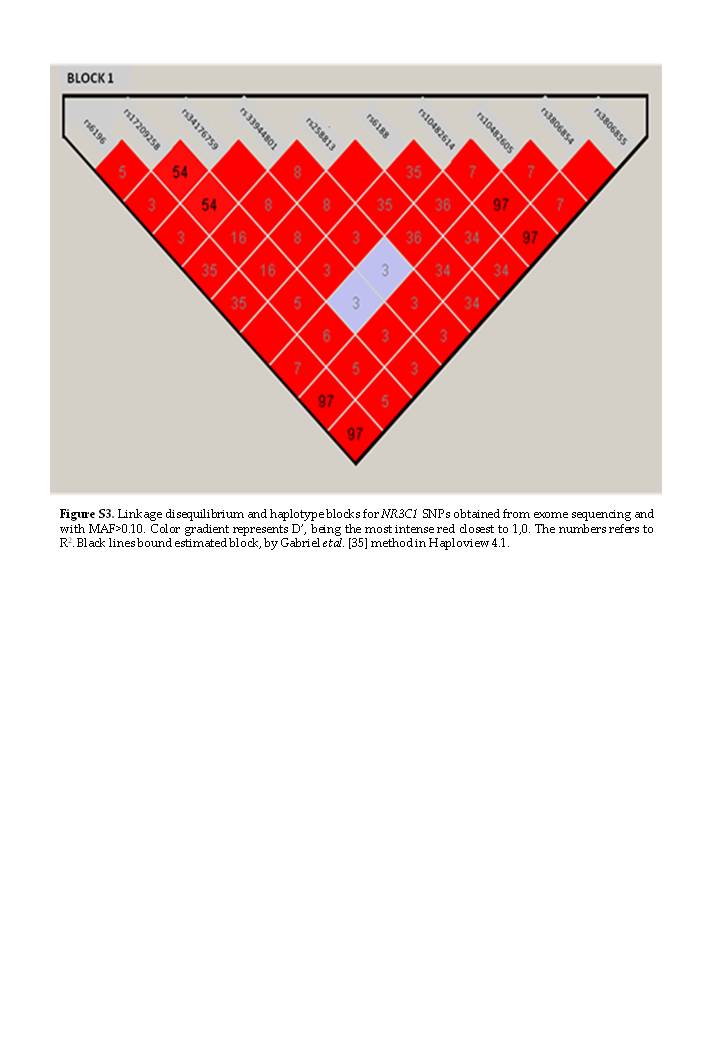

Supplement: Supplementary file 1 [file genes-14-01805-s001.zip › Figure S3_300dpi.jpg]

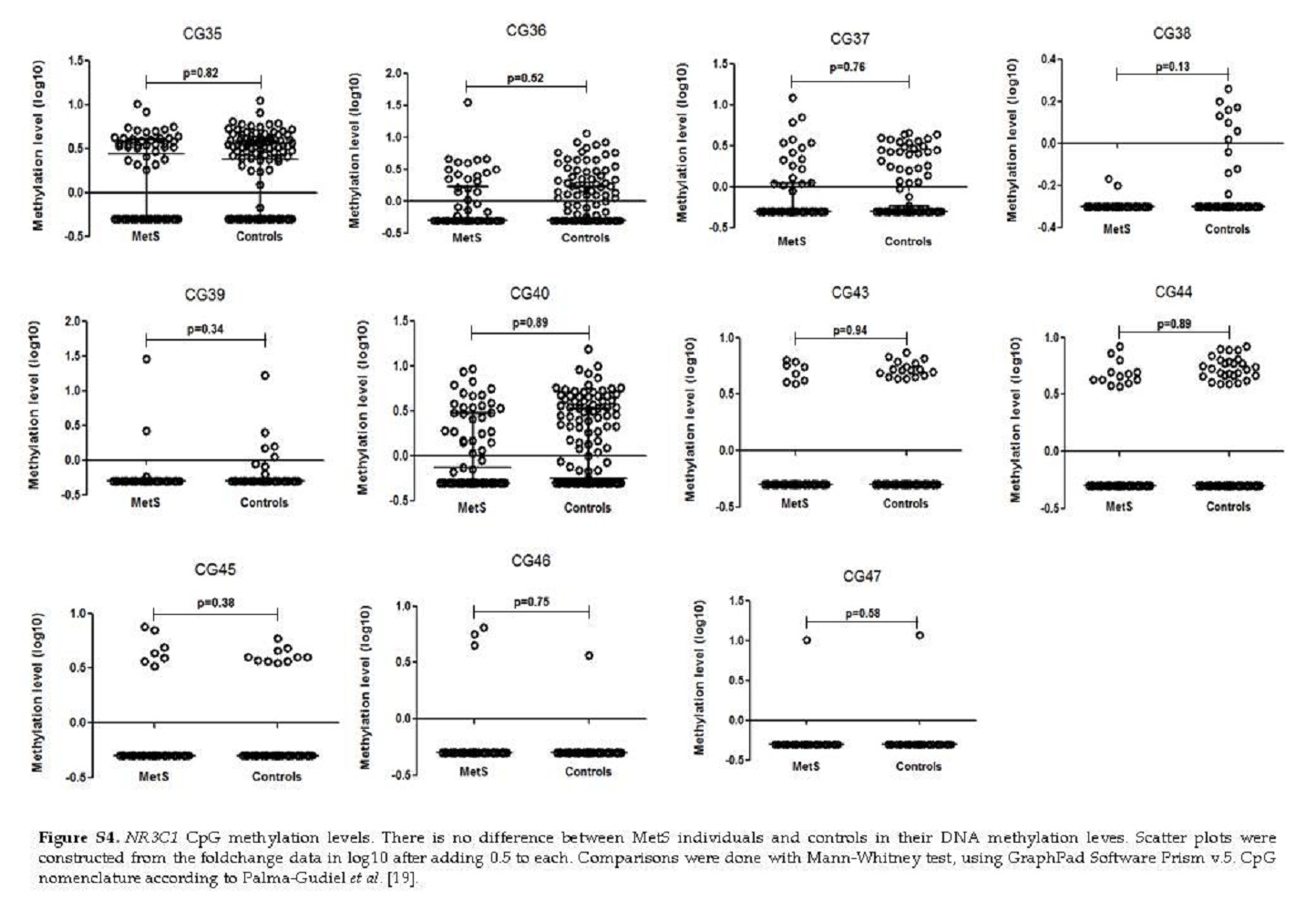

Supplement: Supplementary file 1 [file genes-14-01805-s001.zip › Figure S4_300dpi.jpg]
